# Is schizophrenia disappearing? The rise and fall of the diagnosis of functional psychoses: an essay

**DOI:** 10.1186/s12888-016-1101-5

**Published:** 2016-11-09

**Authors:** Per Bergsholm

**Affiliations:** Department of Psychiatry, District General Hospital of Førde, Box 1000, 6807 Førde, Norway

**Keywords:** Schizophrenia, Psychosis, Functional, Diagnosis, Category, Mood, Affective

## Abstract

**Background:**

The categories of functional psychoses build on views of influential professionals. There have long been four main categories – affective, schizophrenic, schizoaffective/cycloid/reactive/polymorphic, and delusional/paranoid psychoses. The last three are included in “psychotic disorders”. However, this dichotomy and the distinctions between categories may have been over-estimated and contributed to lack of progress.

**Ten topics relevant for the diagnosis of functional psychoses:**

1. The categories of functional psychoses have varied with time, place and professionals’ views, with moving boundaries, especially between schizophrenia and affective psychoses. 2. Catatonia is most often related to affective and organic psychoses, and paranoia is related to grandiosity and guilt, calling in question catatonic and paranoid schizophrenia. Arguments exist for schizophrenia being a “misdiagnosis”. 3. In some countries schizophrenia has been renamed, with positive consequences. 4. The doctrine of “unitary psychosis”, which included abnormal affect, was left in the second half of the 1800s. 5. This was followed by a dichotomy between schizophrenia and affective psychoses and broadening of the schizophrenia concept, whereas affective symptoms were strongly downgraded. 6. Many homogeneous psychoses with mixtures of schizophrenic and affective symptoms were described and related to “psychotic disorders”, although they might as well be affective disorders. 7. Critique of the extensive schizophrenia concept led to, in DSM-III and ICD-10, affective symptoms being exclusion criteria for schizophrenia and acceptance of mood-incongruent psychotic symptoms in affective psychoses. 8. However, affective symptoms are often difficult to acknowledge, diagnosis is often done on the basis of tradition and previous education, and patients’ affect characterized accordingly. 9. DSM-5 is up-dated with separate chapters for catatonia and psychotic symptoms, and removal of the subtypes of schizophrenia. However, time may be running out for categorical psychosis diagnoses, which may be replaced by continuum, spectrum, dimensional and research domain criteria, in line with new biological data 10. This is supported by treatment responses across categories.

**Conclusion:**

The time-consuming works on diagnosis of psychoses may have hampered progress. Chronic mood disorders may appear as schizophrenic or paranoid psychosis, end-stages like heart failure in heart diseases. This underscores the importance of early and optimal treatment of mood disorders.

## Background

The term psychosis is used for mental disorders where the reality testing is grossly impaired [1–3]. Psychotic symptoms manifest as different mixtures of delusions, hallucinations, disorganized thinking and markedly disorganized or catatonic behaviour. Negative symptoms – reduced emotions, interests, will and social participation – are also common, however, unspecific [1, 4, 5]. Organic disturbances in the brain due to infection, neoplasm, vascular disease, toxic substances, autoimmunity, degenerative disease, endocrine disease, metabolic disturbances and head trauma can sometimes provoke psychotic symptoms. When such causes are not present, the term functional psychosis is used [6]. The category diagnosis of functional psychoses builds on the subjective views of influential professionals, and has long included four main groups [6–8]:

1. Affective psychoses or psychotic mood/affective disorders, with the subgroups
  psychotic depression – in depressive (unipolar) or bipolar mood/affective disorder
  psychotic mania or hypomania
  psychotic bipolar mixed state, i. e. psychotic symptoms combined with a mixture of manic/hypomanic and depressive symptoms
2. Schizophrenic psychoses or schizophrenia, with the subgroups
  paranoid schizophrenia
  hebephrenic or disorganized schizophrenia
  catatonic schizophrenia
  If psychotic symptoms are present for less than one month, the term “acute schizophrenia-like psychosis” is used in ICD-10 [9] and “brief psychotic disorder” in DSM-5 [1]. If the total illness duration is less than six months “schizophreniform psychosis” is used in DSM-5. The typical clinical picture consists of bizarre delusions and/or hallucinations when the consciousness is clear and obvious affective symptoms are not present. As soon as the picture deviates from this, it is difficult to differentiate schizophrenia from other functional psychoses.
3. Schizoaffective/cycloid/reactive/polymorphic psychoses,
  i. e. combination of affective and psychotic symptoms, when the latter clearly differ in extension, type or duration from what is usual in affective psychoses.
4. Delusional/paranoid psychoses,
  i. e. psychosis with non-bizarre delusions, usually about persecution or bodily disorder, without other obvious mental symptoms. ICD-10 differentiates between acute transient and persistent delusional disorder.

In the diagnostic manuals the last three categories are included in “psychotic disorders”, because psychotic symptoms are always present when the diagnosis is made. In mood/affective disorders psychotic symptoms are not always present. However, when they are, the disorder is of course a psychotic disorder, and it may be chronic. Therefor, the dividing line between “psychotic disorders” and “affective psychoses” is artificial.

### Ten topics relevant for the diagnosis of psychoses

#### Functional psychoses – moving boundaries between diagnoses

The occurrence and distribution of the groups described above have varied with time, place and the views of professionals. For example, in 1990 Der, Gupta and Murray [10] published an article in Lancet with the heading “Is schizophrenia disappearing?” The first admission rates of patients diagnosed with schizophrenia, schizoaffective and paranoid psychosis in England and Wales had decreased about 50 % from the mid-1960s to the 80s. This was neither due to increase in other diagnoses, nor to more patients being treated ambulatory. The authors assumed that there had been a real fall in the incidence of the psychotic disorders.

Lake and Hurwitz [11] later argued that the distribution of diagnoses has also changed during the last 70 years. They outlined that most psychotic patients were diagnosed with schizophrenia from the 1930s to the 60s. During the 1970s to 90s there was a shift towards an equal distribution of schizophrenia, schizoaffective disorders and affective psychoses, whereas affective psychoses became the most frequent group in the 2000s. The authors pictured that the concepts of schizophrenia and schizoaffective psychosis might disappear, and thought that would be favourable to the patients [8, 11].

#### Schizophrenia – a misdiagnosis?

In DSM-III [12], ICD-10 [9] and DSM-IV [13] there are, as before, three main groups of schizophrenia – paranoid, hebephrenic/disorganized and catatonic. Fink and others [14–16] have long argued that the concept of catatonia should be separated from schizophrenia because it is mostly seen in major mood and organic brain disorders. Lake [17] hypothesized that grandiosity and guilt cause paranoia, so that paranoid schizophrenia is a psychotic mood disorder. Then, there is only hebephrenic or disorganized schizophrenia left. However, Lake [8] later reasoned extensively for schizophrenia being a “misdiagnosis”. He argues that the majority of patients he was educated to believe had schizophrenia suffered from psychotic mood disorders, the rest having subtle and non-discovered organic causes. He has summarized the opinions of influential professionals [8]. Several share his view, and my own experience is that he may be right in most of his arguments.

#### The term schizophrenia – time for change?

Schizophrenia has become a stigmatizing term. Therefore, in Japan (2002) and South Korea (2012) schizophrenia has been renamed, which has reduced the stigma and improved the communication with the patients [18–20]. In the minds of young Japanese schizophrenia was strongly associated with “criminal”, and this was reduced with the new name [20]. Others, too, think that time is in for replacement of the term [21], for one thing because it leads the thought away from other aspects than treatment with antipsychotics. Especially, this concerns treatment of mood disturbance, which may be masked by psychotic symptoms [22]. Mood and thinking are always associated, and mood disturbance is central to psychosis. This has been acknowledged from the antiquity. However, from the end of the 1800s this was overshadowed by a dominating emphasis on thought disturbance. As will be delineated, history has shown that this has been unfavourable, because it led to underestimation of emotional life and mood [8].

#### Mania, melancholia, unitary psychosis, several psychoses – from the antiquity to the 1900s

The great diagnostic categories of the past originated in the Greek antiquity, and the early conceptions of mania and melancholia included largely all the conditions described as functional psychoses from the 1800s [7, 23]. From the second century AD it was known that mania and melancholia often were two aspects of the same illness, and from the 1600s manic-melancholic (later manic-depressive or bipolar] mixed states were described [23]. Some common pathology was assumed to underlie all functional psychoses, and in the first half of the 1800s the doctrine of “unitary psychosis” emerged [2, 24].

Thus, disturbed mood and energy had been viewed as a central part of functional psychoses ever since the antiquity. However, from the second half of the 1800s the idea of unitary psychosis receded into the background. This happened after Wilhelm Griesinger (1817–68) changed his view and together with Ludwig Snell (1817–92) and Bénédict Morel (1809–73) strongly argued for other psychotic illnesses in addition to psychosis in melancholia, mania and mixed states, although chronic and disabling forms of these were well known from the antiquity [8, 24]. Karl Ludwig Kahlbaum (1828–99) and his pupil Ewald Hecker (1843–1909) described in 1860-70 a psychosis variant, hebephrenia, in young people who’s childhood development most often had been somewhat slow [25]. However, although they thought there might be a distinct etiology, the starting point was mood disorder: The illness progressed “from melancholia, to mania, to confusion, and then to dementia” [14, 26].

Kahlbaum also described catatonia [27], i. e. severe motor disturbance in psychoses, from complete passivity (stupor and mutism) to strange movements, postures and marked restlessness (agitation) [14, 16]. However, “this little monograph was not, of course, an attempt to describe a new disease but to put order in the confused field of melancholia attonita” [7]. Other types of psychosis were also described, especially periodic and circular insanity, amentia/confusion psychosis, and dementia paranoides.

Thus, at the end of the 1800s many professionals thought that there were several different psychotic illnesses, whereas others, as Gustav Specht (1860–1940), continued to think that all functional psychoses derive from an abnormal affect [8].

#### Dementia praecox/schizophrenia and manic-depressive illness – a dichotomy

A turning point came in 1899 when Emil Kraepelin (1856–1926) presented his dichotomy. He grouped hebephrenia, catatonia, and dementia paranoides (which he himself described) in dementia praecox. Melancholia, mania, manic-melancholic mixed states, periodic and circular insanity, amentia/confusion psychosis and paranoia were included in manic-depressive insanity [6, 28]. The Kraepelinian concept of manic-depressive illness was broad. His concept of dementia praecox was narrow due to the criterion of a course leading to psychic invalidity. Melancholic and manic syndromes were neither included nor excluded [6].

As time went by Kraepelin was in doubt of his dichotomy, and in 1920 he wrote: “It is becoming increasingly clear that we cannot distinguish satisfactorily between these two illnesses and this brings home the suspicion that our formulation of the problem may be incorrect” [8, 29]. However, the self-criticism of the older Kraepelin was little noticed, whereas the dichotomy of the younger one was appealing in its simplicity. There was now in the minds of professionals established a mental “firewall” between dementia praecox and manic-depressive insanity [14].

In 1911 Paul Eugen Bleuler (1857–1939) introduced the term schizophrenia to replace dementia praecox, markedly broadened the concept and reinforced the “firewall”. He described “basic or fundamental” disturbances, which he meant were pathognomonic to schizophrenia, whereas delusions and hallucinations were “accessory” symptoms [6]. However, his “basic or fundamental” disturbances overlap with the later concept of “negative symptoms” [5], which may also be due to depression [4, 5, 8, 30], and include “formal thought disorders”, which may also be due to the rich associative thinking in bipolar disorder [8]. Bleuler included the possibility of melancholic, manic and catatonic syndromes in his criteria [6], and explicitly stated that the diagnosis of manic-depressive insanity should only be made after exclusion of schizophrenia [31]. The expanded schizophrenia concept of Bleuler is illustrated by a quotation from his 1924 textbook [32]:

“Under schizophrenia are included many atypical melancholias and manias of other schools (especially nearly all ‘hysterical’ melancholias and manias), most hallucinatory confusions, much that is elsewhere called amentia (…), a part of the forms cosigned to delirium acutum, motility psychosis of Wernicke, primary and secondary dementias without special names, most of the paranoias of the other schools, especially all hysterically crazy, nearly all incurable ‘hypochondriaes’, some ‘nervous people’ and compulsive and impulsive patients.”

In the years 1939-59 the “firewall” was further strengthened by Kurt Schneider (1887–1967). He listed “first rank” symptoms – hallucinations and delusions he meant were specific to schizophrenia, especially the experience of being influenced and talked to or about [6]. Thus, he emphasized what Bleuler had relegated to accessory symptoms, and wrote: “When any of these modes of experience is undeniably present, and no somatic illness can be found, we may make the decisive clinical diagnosis of schizophrenia” [31, 33]. He included depressive and euphoric mood changes in the “second rank” symptoms. Neither Bleuler nor Schneider included the course criterion of Kraepelin, whereas both based the differential diagnosis on the hierarchical principle of Karl Jaspers (1883–1969): After organic symptoms came schizophrenic/psychotic symptoms, which came before affective and neurotic/personality symptoms [6]. The importance of affective symptoms was strongly downgraded.

Sigmund Freud’s (1856–1939) hypotheses offered explanations and hope for treatment. In his review of catatonia, Max Fink wrote: “An image of dementia praecox as a brain disease was replaced by an image of disorganization induced by childhood experience and memories, best relieved by individual psychoanalysis, a philosophy enthusiastically adopted by Paul Eugene Bleuler” [14]. Therefore, it was important to make an early diagnosis. For example, in a textbook Lewis & Piotrowski (1954) wrote that “even a trace of schizophrenia is schizophrenia” [31]. All this made the concept of schizophrenia extensive and unclear – as is still the case [8].

#### Psychoses between schizophrenia and affective psychosis

Several influential psychiatrists tried to compensate for the “firewall” by defining disorders with a mixture of schizophrenia-like and affective symptoms [6], and several terms appeared for overlapping syndromes.

The American psychiatrist Jacob Kasanin (1897–1946) introduced in 1933 the term schizoaffective psychosis with reference to nine patients who had previously been diagnosed with dementia praecox/schizophrenia [6, 34]. Lake [8] believes that these patients today would have got the diagnosis of psychotic mood disorder. In DSM-IV and DSM-5 schizoaffective disorder is used when delusions or hallucinations last at least two weeks longer than obvious affective symptoms. In ICD-10 the diagnosis depends on an “approximate’balance’ between the number, severity, and duration of the schizophrenic and affective symptoms”. From this, it is obviously difficult to differentiate between schizoaffective and affective psychosis.

The Norwegian psychiatrist Gabriel Langfeldt (1895–1983) followed in the 1920–30s a similar patient group, which he labelled schizophreniform psychosis [6]. However, a later follow-up examination of the case records indicated that most of his patients had suffered from affective disorders [35]. The term existed in ICD-8 (1967) and ICD-9 (1978), but was removed in ICD-10 (1993), whereas it remains in the DSM system.

The German psychiatrists Karl Kleist (1879–1960) and Karl Leonhard (1904–88) described similar syndromes in the first and midst of the twentieth century. They launched the term cycloid psychosis due to the cyclic course. From its predecessors motility and anxiety psychoses of Wernicke (1848-1905) they delineated three overlapping forms: motility psychosis, confusion psychosis and anxiety-elation-psychosis [6, 36–38]. These names point to disturbed motor activity (from severe agitation to immobility, i. e. like catatonia), confusion and emotional chaos as hallmarks. Postpartum psychosis became the “flagship” for this diagnosis [39]. The Italian-Swedish psychiatrist Carlo Perris (1928–2000) extended the works of Kleist and Leonhard [6, 40], and underscored the importance of response to electroconvulsive therapy and lithium.

Corresponding syndromes were in Denmark and Norway often named psychogenic or reactive psychosis [41, 42], the latter once being the most frequent diagnosis of psychosis in Norway [6], due to the impact of psychiatrist Niels Retterstøl (1924–2008). The most severe forms equalled a syndrome with many names: Bells mania, delirium grave, delirium acutum, delirious mania, acute deadly psychosis and lethal/malignant catatonia [16, 43–45]. Thus, the Danish psychiatrist Erik Strömgren (1909-1993), in a chapter on reactive psychosis, wrote [46]: “If there is the slightest sign to the condition developing into the life-threatening delirum acutum (…), electroshock treatment is indicated.”

In ICD-10 [9] the new term acute polymorphic psychosis was chosen for syndromes as above, due to the diverse and often abrupt changing symptoms. It is assigned to chapter F2 psychotic disorders. However, it has much in common with affective psychosis, and can be considered a subgroup of bipolar disorder as well. Thus, the Swedish psychiatrist Jan-Otto Ottosson, in his textbook from 1983 to 2015, uses cycloid syndrome as a synonym for polymorphic psychosis, and classifies it as a variant of bipolar disorder [47].

The terms paranoid psychosis and paranoid schizophrenia are often used interchangeably, the latter preferentially later in an illness course or if hallucinations are present [6]. However, one should always suspect hidden mood disorder behind persecutory and bodily delusions [17, 22].

#### Critics of the extensive schizophrenia concept – towards modern diagnosis

The extensive concept of schizophrenia was increasingly criticized, particularly in USA. For example, Pope Jr and Lipinski Jr [31] wrote: “… most so-called schizophrenic symptoms, taken alone and in cross section, have remarkably little, if any, demonstrated validity in determining diagnosis, prognosis, or treatment response in psychosis… overreliance on such symptoms alone results in overdiagnosis of schizophrenia and underdiagnosis of affective illnesses, particularly mania. This compromises both clinical treatment and research.”

In 1980 this critic was taken account of in DSM-III [12]. The hierarchy of Jaspers was reversed by making depressive and manic/hypomanic symptoms exclusion criteria for schizophrenia, and it was emphasized that mood incongruent psychotic symptoms, as delusions and hallucinations about persecution and influence, could occur in affective disorders.

However, outside USA the diagnostic system was ICD-9 from 1978, which for schizophrenia was identical with ICD-8 from 1969 [6], building on the criteria of Bleuler and Schneider. For example, in 1981 the Norwegian health director wrote [48]: “It is natural to emphasize schizophrenic basic symptoms in the differential diagnosis, whereas symptoms like melancholiform depression or maniform excitement carry less weight in favour of the diagnosis of manic-depressive psychosis”. In 1993, however, ICD-10 [9], too, included affective syndromes as exclusion criteria for schizophrenia.

#### Affective symptoms are often difficult to acknowledge

The clinician’s education, experience and preconception are crucial to diagnosis [49]. Thus, referring to studies on the specificity of Bleulerian symptoms, Andreasen and Akiskal [30] wrote: “Typically, clinicians decided that the patient had schizophrenia or depression and then characterized his affect accordingly.” When psychologist Arnhild Lauveng [50] is memorizing to “… the big greyness … how the world lost its colours and I was afraid of being dead”, she thinks this must be “affective flattening” and “prodromal syndrome”, phrases she has learned to be related to schizophrenia. However, such symptoms indicate severe depression as well, and “affective flattening” is often pronounced in mood disorders [4, 5]. “Prodromal syndrome” is not specific to schizophrenia, either. It may develop in different directions, most often to a non-psychotic mood disorder [51]. Lauveng [50] also describes a phase compatible with hypomania prior to her psychosis, indicating a mood disorder.

Pathologic depression is not common sadness, but mental pain and often also physical pain [22], with a distinct quality that cannot be fully defined [7, 52]. Consequently, “the clinical disorders of affect struggled for recognition”, and those afflicted are often “unable to behave as a rational observer” [7]. The patient may deny depression and the clinician may end up characterizing the patient’s mood as “neutral”. The patient doesn’t necessarily look like being depressed, and might even appear lively if he/she has a bipolar disorder or temperament. Depression may become more obvious when psychotic symptoms diminish, however, may then be explained as “post-psychotic depression”. Thoughts and emotions are always intermingled, and being psychotic with “neutral” mood seems anti-intuitive.

Many words and phrases have been used in diagnostic criteria for pathological depression – sad, dysphoric, depressed, blue, low, down in the dumps, despondent, hopeless, irritable, fearful, worried, anxious, discouraged, don’t care, loss of pleasure/enjoyment, loss of interest [6]. However, none is fully adequate. The “Vienna research criteria” of Berner [6] are based on objective signs – changes in affectivity, emotional resonance, drive, and biorhythm – resembling “negative symptoms”. Anhedonia is central to depression [7], as are impaired self-respect, self-esteem, self-love, and self-preservation [52]. Suicide is often the only way out of frantic hopelessness, emotional pain, ruminative flooding and (near) psychotic somatization [22, 53].

#### Down the road – DSM-5, continuum, spectrum, dimensions, genetics, pathology

In ICD-10 catatonia is mentioned briefly under “Other nonorganic psychotic disorders”, however, without diagnostic codes. In DSM-IV it was possible to add catatonia as a specifier to other diagnoses than schizophrenia, but again without codes. Therefore, this had little impact, because “in some way disease does not exist until we have agreed that it does – by perceiving, naming, and responding to it” [54]. DSM-5 [1], however, seems to have been influenced by Fink’s group [14] – catatonia has got its own chapter with diagnostic codes (although placed under the heading “Schizophrenia spectrum disorders and other psychotic disorders”). This is important because catatonia often requires other treatment options than schizophrenia and other psychoses [16, 55, 56]. Lake and Hurwitz [8, 11, 17], too, seems to have been listened to – the subgroups of schizophrenia have been removed in DSM-5. Moreover, psychotic symptoms are described in a separate chapter independent of specific categories (although, this too, under “Schizophrenia spectrum disorders and other psychotic disorders”). Thus, there are no longer any psychotic symptoms specific of schizophrenia or other disorders. Affective/mood syndromes still have priority in the differentiation between affective psychoses and so-called psychotic disorders or non-affective psychoses.

The works on diagnosing psychoses seems to have been of doubtful value. “The ‘Babel of differing formulations in psychiatric diagnosis’ (Brockington et al., 1978) prompted the elaboration of compromise classification systems … The compromise character of these large diagnostic systems makes them unsuitable for psychiatric research”, Berner et al. [6] wrote in 1983/1992. They argued for the polydiagnostic approach, however, neither this approach seems to have contributed much [57]. Kraemer et al. [58] underlined that:” … efforts to improve reliability by simply redefining diagnostic categories are bound to fail”. Johannessen and McGorry [51] argued that “mental disorders in general, and the psychotic disorders in particular, are not static, sharply defined illnesses … but rather disorders that overlap (dimensions) and develop in stages … the current diagnostic systems define an end-state syndrome … time is running out for the more established concepts such as schizophrenia.” The DSM-5 Psychotic Disorders work group wrote [59]: “There is increasing evidence that the categorical diagnosis of schizophrenia and other psychotic disorders contributes to lack of progress.”

Accordingly, brain imaging [60–62] and morphological [63, 64] studies show overlapping results for schizophrenia and bipolar disorder. There is also a marked genetic correlation between schizophrenia, bipolar disorder and major depressive disorder [65], although there are likely also genetic loci specific to each disorder [66], and the clinical variations are innumerable. Tesli et al. [67] find support for a “floating continuum model rather than distinct diagnostic entities.” They include affective psychoses in “one common broad psychosis spectrum”, consistent with the many common features in the descriptions of different psychoses [8]. Timothy Crow [68, 69] has for more than 30 years argued for “a continuum extending from unipolar, through bipolar affective illness and schizoaffective psychosis, to typical schizophrenia, with increasing degrees of defect.”

Van Os and Kapur [70] suggested rating of the five dimensions psychosis (positive symptoms), negative symptoms, neurocognitive alterations, mania and depression, in addition to category diagnosis. In my opinion, motor and vegetative symptoms should be included as separate dimensions. A new approach, The Research Domain Criteria (RDoC), “is already freeing investigators from the rigid boundaries of symptom-based categories”, according to Insel [71] at the National Institute of Mental Health. RDoC implies relating neurobiological findings (genes, molecules, cells, circuits, physiology) to behaviour, self-report and treatment effects independent of predefined categories, to find new constellations or dimensions with better validity [64].

#### Treatment – early and across diagnostic categories

Various treatment options may be indicated across diagnostic categories. Differences in response rate between groups cannot be relied upon when treating an individual patient. Although antipsychotics are usually first choice among drugs in psychosis, they are often insufficient [72]. Moreover, they may induce an “anti-joy-of-life” effect [73] or deficit syndrome resembling negative symptoms [74], and in the long run carry the risks of tardive psychosis [22, 75] and lower functional remission rate [76, 77]. Antidepressants may reduce negative symptoms [78] and suicide in schizophrenia [79, 80], and in one study reduced transition to psychosis in high-risk subjects more than antipsychotics [81], consistent with psychotic experiences being a marker foremost of affective dysregulation [82]. Response to lithium may only be determined by trial and error across diagnostic borders [22, 83, 84], in accordance with Gualtieri [85]: “Nevertheless, in people with mental retardation, as in other classes of patients, success may be found with lithium when no other drug has been successful and in unusual conditions in which a likely response can hardly be predicted.” Electroconvulsive therapy may be indicated in mostly all severe psychotic states, regardless of diagnostic label [86–88].

Chronicity and suicide justify the classical question of quality control: “Was the right thing done, and was it done right?” [89]. In spite of limited efficacy of available treatment options, all methods must be considered as soon as possible, because psychotic or near psychotic states in general are more serious than the risks of treatment trials [90, 91]. Diagnostic labels for psychotic disorders, especially schizophrenia and paranoid psychosis, may have been obstacles to other treatment options than antipsychotics [49, 72].

## Conclusion

The concept of schizophrenia may disappear and the diagnosis of psychoses characterized as a failure. However, there are signs of rebuilding. The “firewall” between psychotic and affective disorders should be replaced by descriptions of dimensions and illness spectra. Phrases like “neutral” and “flat” affect/mood should be avoided due to the difficulty in acknowledging pathological mood, which may be masked as “negative” symptoms or deviating associations.

Chronic, often sub-optimally treated, severe/psychotic mood disorder may appear as schizophrenia or paranoid psychosis [8, 22]. Such syndromes often represent end-stages [51], like heart failure being the end-stage of different heart diseases. All available treatments must be tried to stop a development towards this stage. This strengthens the importance of optimal treatment of mood disorders, which may be the most important cause of schizophrenia and other functional psychoses.

## Abbreviations

DSM: Diagnostic and statistical manual
ICD: International classification of diseases
